# 

**Published:** 1882-09

**Authors:** 


### BOOKS RECEIVED.

**Transactions of the Medical and Chirurgical Faculty of the State of Maryland.** Eighty-fourth annual session held at Baltimore, Md., April, 1882.

---

**A Study of Rupture of the Bladder,** with some experiments bearing upon the closure of the vesical wound. By ALEX. W. STEIN, M. D., of New York. Reprint. New York: Gustav E. Stechert, 766 Brodway. 1882.

